# A Role of TDIF Peptide Signaling in Vascular Cell Differentiation is Conserved Among Euphyllophytes

**DOI:** 10.3389/fpls.2015.01048

**Published:** 2015-11-26

**Authors:** Yuki Hirakawa, John L. Bowman

**Affiliations:** ^1^School of Biological Sciences, Monash UniversityMelbourne, VIC, Australia; ^2^Institute of Transformative Bio-Molecules (WPI-ITbM), Nagoya UniversityNagoya, Japan; ^3^Section of Plant Biology, University of California, DavisDavis, CA, USA

**Keywords:** CLE peptides, plant evo-devo, LRR-RLKs, plant vascular development, vascular plants, non-model organism

## Abstract

Peptide signals mediate a variety of cell-to-cell communication crucial for plant growth and development. During *Arabidopsis thaliana* vascular development, a CLE (CLAVATA3/EMBRYO SURROUNDING REGION-related) family peptide hormone, TDIF (tracheary element differentiation inhibitory factor), regulates procambial cell fate by its inhibitory activity on xylem differentiation. To address if this activity is conserved among vascular plants, we performed comparative analyses of TDIF signaling in non-flowering vascular plants (gymnosperms, ferns and lycophytes). We identified orthologs of TDIF/CLE as well as its receptor TDR/PXY (TDIF RECEPTOR/PHLOEM INTERCALATED WITH XYLEM) in *Ginkgo biloba, Adiantum aethiopicum*, and *Selaginella kraussiana* by RACE-PCR. The predicted TDIF peptide sequences in seed plants and ferns were identical to that of *A. thaliana* TDIF. We examined the effects of exogenous CLE peptide-motif sequences of TDIF in these species. We found that liquid culturing of dissected leaves or shoots was useful for examining TDIF activity during vascular development. TDIF treatment suppressed xylem/tracheary element differentiation of procambial cells in *G. biloba* and *A. aethiopicum* leaves. In contrast, neither TDIF nor putative endogenous TDIF inhibited xylem differentiation in developing shoots and rhizophores of *S. kraussiana*. These data suggest that activity of TDIF in vascular development is conserved among extant euphyllophytes. In addition to the conserved function, via liquid culturing of its bulbils, we found a novel inhibitory activity on root growth in the fern *Asplenium* × *lucrosum* suggesting lineage-specific co-option of peptide signaling occurred during the evolution of vascular plant organs.

## Introduction

Recent advances in biochemical, genetic and bioinformatic analyses have unveiled the importance of peptide hormones in plant growth and development (Matsubayashi, 2014). CLE (CLAVATA3/EMBBRYO SURROUNDING REGION-related) peptides are a class of peptide hormones involved in an array of plant developmental processes including shoot apical meristem maintenance, vascular cell differentiation and stem cell maintenance, root meristem maintenance, development of embryo and endosperm, autoregulation of nodulation, lateral root responses to nutrient conditions and pollen viability (Fletcher et al., 1999; Brand et al., 2000; Hirakawa et al., 2008; Okamoto et al., 2009; Kondo et al., 2011; Fiume and Fletcher, 2012; Depuydt et al., 2013; Endo et al., 2013; Araya et al., 2014). Typical CLE proteins contain an N-terminal signal peptide and a CLE peptide motif near the C-terminus which are intervened by non-conserved variable region. The mature signaling peptides are produced from the CLE peptide motif as 12–13 amino acid peptides containing proline hydroxylation and glycosylation via post-translational processing (Ito et al., 2006; Kondo et al., 2006; Ohyama et al., 2009; Ogawa-Ohnishi et al., 2013). Secreted CLE peptides are perceived by receptors residing in target cell membranes to mediate intercellular signaling. Based on the bioactivity and receptor specificity, two major subgroups can be recognized in the CLE peptide family: here we call R-type CLE and H-type CLE (Ito et al., 2006; Strabala et al., 2006; Kinoshita et al., 2007; Whitford et al., 2008; Ohyama et al., 2009). Each has a characteristic amino acid residue (arginine or histidine) at the N-terminus of the peptide. The R-type CLE includes CLV3 (CLAVATA3), which plays a significant role in the maintenance of shoot apical meristem in *Arabidopsis thaliana*, while the H-type CLE includes TDIF, an important regulator of vascular cell differentiation (Brand et al., 2000; Schoof et al., 2000; Ito et al., 2006; Hirakawa et al., 2008). Peptides in each subgroup are perceived through specific receptors of LRR-RLK family, CLV1 (CLAVATA1)/BAM (BARELY ANY MEISTEM) or TDR/PXY (TDIF RECEPTOR/PHLOEM INTERCALATED WITH XYLEM; Clark et al., 1995; Brand et al., 2000; DeYoung et al., 2006; Fisher and Turner, 2007; Hirakawa et al., 2008; Ogawa et al., 2008; Shinohara et al., 2012). The TDIF-TDR pair mediates a phloem-derived signal that inhibits differentiation of procambial cells into xylem cells, which is important during secondary growth of vasculature in *A. thaliana* floral stems (Hirakawa et al., 2008, 2010; Whitford et al., 2008; Etchells and Turner, 2010).

The CLE family is conserved throughout land plants although functional paralogs are not precisely characterized except in angiosperms. Similar to many other gene families of developmental regulators, the number of genes seems lower in early diverging taxa such as the bryophytes and lycophytes (1 and 15 sequences are reported for *Physcomitrella patens* and *Selaginella moellendorffii*, respectively), compared to the number found in flowering plant species such as *A. thaliana*, which possesses 32 CLE genes (Jun et al., 2008; Oelkers et al., 2008; Miwa et al., 2009). Thus, the expansion of the CLE gene family and subsequent neofunctionalization may have played important roles in the evolution of land plant development, particularly in the vascular plant lineages.

In this study, we performed evolutionary and functional comparative analyses of TDIF/H-type CLE peptides among major lineages in vascular plants—angiosperms, gymnosperms, ferns and lycophytes.

## Materials and methods

### Database search for orthologs of TDIF and TDR genes

Nucleotide or protein sequences corresponding to the CLE peptide motif of *A. thaliana* CLE41/At3g24770 (His^87^ to Asn^99^), the CLE peptide motif of *P. patens* CLE1/CLE170/XM_001752838 (Arg^136^ to Asn^147^) and the kinase domain of *A. thaliana* TDR/At5g61480 (Gly^726^ to Leu^997^) were used as queries for database searches. BLAST searches were performed against the SRA (Sequence Read Archive) and oneKP (one thousand plants, http://www.onekp.com/) databases, focusing on EST data for gymnosperms, ferns and lycophytes, as well as Genbank transcript data (de Vries et al., 2015; Vanneste et al., 2015). Each of the obtained sequences was manually validated to determine whether it encodes a complete protein containing an N-terminal signal peptide by SignalP (http://www.cbs.dtu.dk/services/SignalP/).

### RNA extraction and cDNA synthesis

Total RNA was extracted from immature leaves/fronds of *Ginkgo biloba, Adiantum aethiopicum*, and *Selaginella kraussiana*, using the RNeasy Plant Mini Kit (Qiagen) with modifications: adding 1% polyethylene glycol into the lysis buffer (RLC buffer) and repeating an extra EtOH buffer (RPE buffer) wash. Reverse-transcription (RT) reactions were performed against the extracted total RNA using either the Super Script III (Life Technologies) or the SMART RACE cDNA Amplification Kit (Clontech) according to the manufacturers' instructions.

### Degenerate PCR and smart-race PCR

Degenerate primers were designed based on the conserved amino acid sequences within the CLE peptide motif for TDIF or the kinase domain for TDR (Table S2). SMART-RACE PCR was performed using SMART RACE cDNA Amplification Kit (Clontech) with primers described in Table S2. Genbank accession numbers for the obtained sequences are KT343281–KT343287 as indicated in Table S2.

### Phylogenetic analysis

The sequences were first aligned in Clustal X. We excluded ambiguously aligned sequence to produce an alignment of 253 amino acid characters. Phylogenetic analyses were performed using MrBayes 3.2.1 (Huelsenbeck and Ronquist, 2001) and analyses were run for 500,000 generations, which was sufficient for convergence of the two simultaneous runs of each analysis. Convergence was assessed by visual inspection of the plot of the log likelihood scores of the two runs calculated by MrBayes (Gelman and Rubin, 1992). Character matrix and command files used to run the Bayesian phylogenetic analysis are provided in Data Sheet S1.

### Plant culture and peptide treatment

Immature *G. biloba* leaves, immature *A. aethiopicum* fronds and *S. kraussiana* shoots of 5 mm in length were excised and surface sterilized in sterilization solution (1% sodium hypochlorite and 0.1% TritonX-100) for 3–5 min, then washed 4 times with water. For *Asplenium* × *lucrosum* bulbils, all visible leaves were detached and the sterilization was performed for 15 min. All plant samples were cultured in half-strength MS liquid medium containing 1% sucrose and 0.05% MES (pH 5.8) at 22°C under continuous light without shaking. The bulbils were transferred to new liquid culture medium every 3 weeks. In the peptide treatment assays, plant samples of similar size/developmental stage were collected for the replicate of control and peptide-treatment samples. TDIF, (HEVHypSGHypNPISN), SkCLE1 (HSVHypSGHypNPVGN), and SkCLE1L (HSVHypSGHypNPVGNSLPG) peptides were chemically synthesized with >95% purity (Operon Biotechnologies). All experiments were replicated at least three times.

### Observation of vasculature

Leaves/fronds were fixed in a 1:3 mixture of acetic acid/ethanol, washed with water and mounted in a mixture of chloral hydrate/glycerol/water (8:1:2). For sectioning, samples were fixed in FAA solution (50% ethanol: 10% formalin: 5% acetic acid in water) and embedded using the JB-4 embedding kit (Polysciences) according to the manufacturer's instructions. Blocks were sectioned at 3 μm thick and the sections were stained with 0.05% toluidine blue and observed with a Zeiss Axioskop microscope.

## Results

### TDIF genes in vascular plants

TDIF genes in non-flowering vascular plants were identified by searching the Genbank and 1 KP databases using the amino acid sequence of TDIF, HEVPSGPNPISN, as a query. This revealed TDIF-like gene transcripts in many gymnosperms and ferns. For example, CLE peptide motifs identical to TDIF were found in *Picea sitchensis, Pseudotsuga menziesii, Taxus baccata, Sequoia sempervirens, Gnetum gnemon, G. biloba, Equisetum giganteum* (Table S1). In the transcript data for the lycophyte *Huperzia squarrosa*, we found two H-type CLE and an R-type CLE sequences although we could not find CLE peptide motifs identical to TDIF in lycophyte data. These sequences were also different from any of the five H-CLE sequences of *S. moellendorffii*, encoded by SmCLE12-15 (Miwa et al., 2009). In the moss *P. patens*, a CLE gene has been reported and designated as CLE170/PpCLE1 (Oelkers et al., 2008; Miwa et al., 2009). Using the CLE motifs of PpCLE1 in addition to TDIF as queries, we found additional 5 CLE sequences in Genbank transcript database (designated as PpCLE2 to PpCLE6; Table S1). However, all encode R-type CLE genes and no additional H-type CLE gene was detected.

**Figure 1:**
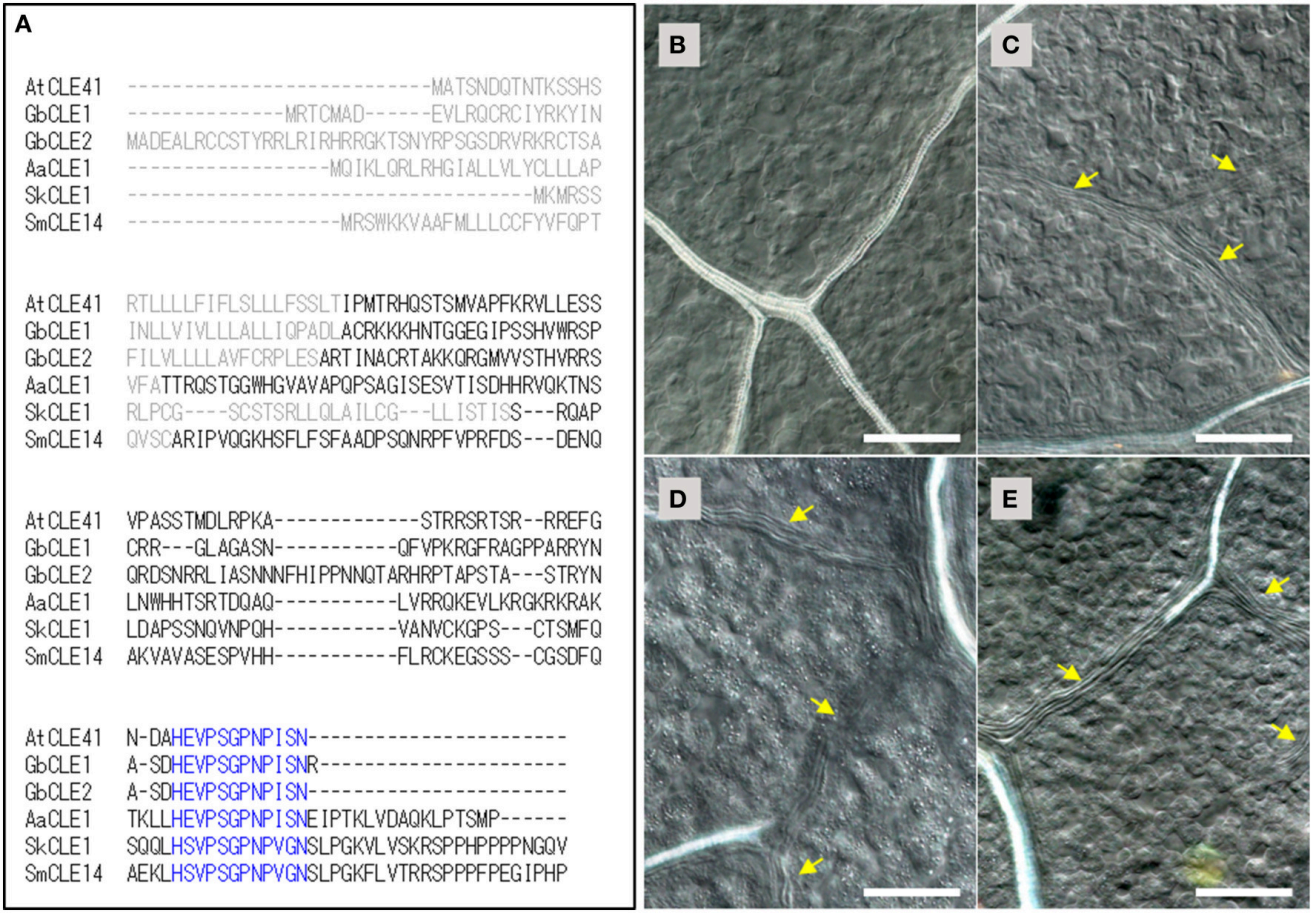
**TDIF/H-type CLE genes in land plants and the bioactivities of CLE peptides in ***Arabidopsis thaliana*****. **(A)** Alignment of deduced primary sequences for TDIF genes identified in this study (*Ginkgo biloba* CLE1, *Adiantum aethiopicum* CLE1, *Selaginella kraussiana* CLE1) with *A. thaliana* CLE41 and *Selaginella moellendorffii* CLE14. Gray and blue texts indicate signal peptide and the 12 amino-acid CLE peptide motifs, respectively. **(B–E)** Effects of peptides in *A. thaliana* plants grown for 10 days in liquid medium containing no additional peptide **(B)**, 5 μM TDIF **(C)**, 5 μM SkCLE1 peptide **(D)**, or 5 μM SkCLE1L peptide **(E)**. Yellow arrows indicate veins without visible xylem vessels. Scale bars: 100 μm.

We next isolated TDIF orthologs from cDNA of *G. biloba, A. aethiopicum*, and *S. kraussiana* by degenerate PCR and RACE PCR. For *G. biloba* TDIF genes (*GbCLE1* and *GbCLE2*), two partial sequences obtained in the BLAST search were used to design primers for RACE-PCR. *GbCLE1* and *GbCLE2* sequences exhibit a typical CLE protein organization: an N-terminal signal peptide, a CLE peptide motif near or at the C-terminus and an intervening non-specific region (Figure 1A). In *A. aethiopicum* and *S. kraussiana*, amplification of CLE peptide sequences was performed by degenerate SMART-RACE PCR with the primers corresponding to the first several amino acids in the CLE peptide motif and 3′-end universal primers for SMART-RACE PCR (Table S2). We could detect single genes in the two species, namely *AaCLE1* and *SkCLE1*. The SkCLE1 sequence was highly similar to CLE14 of *S. moellendorffii*. Both *AaCLE1* and *SkCLE1* had the typical CLE protein configuration (Figure 1A).

The primary sequences of the CLE peptide motif of *AtCLE41/-44, GbCLE1, GbCLE2*, and *AaCLE1* were identical while *SkCLE1* has a few substitutions relative to the other sequences (Figure 1A). As these substituted residues are reported to be not essential for bioactivity in the xylem cell differentiation assay (Ito et al., 2006), SkCLE1 peptide would be predicted to possess the TDIF-like bioactivity in angiosperms. In *A. thaliana*, exogenous TDIF suppresses xylem differentiation when plants are grown in liquid culture medium (Figures 1B,C; Hirakawa et al., 2008), and indeed, SkCLE1 peptide (H-S-V-Hyp-S-G-Hyp-N-P-V-G-N) exhibited a similar bioactivity (Figure 1D). A longer CLE peptide, SkCLE1L (H-S-V-Hyp-S-G-Hyp-N-P-V-G-N-S-L-P-G), was also examined since C-terminal cleavage of the SkCLE1 peptide might occur either at the homologous position (Asn^82^-Ser^83^) or between the Gly^86^and Lys^87^, catalyzed by proteases like the *A. thaliana* SOL1 carboxypeptidase (Tamaki et al., 2013). The SkCLE1L peptide showed a similar bioactivity as SkCLE1 and TDIF peptides in *A. thaliana* (Figure 1E).

### TDR genes in vascular plants

**Figure 2:**
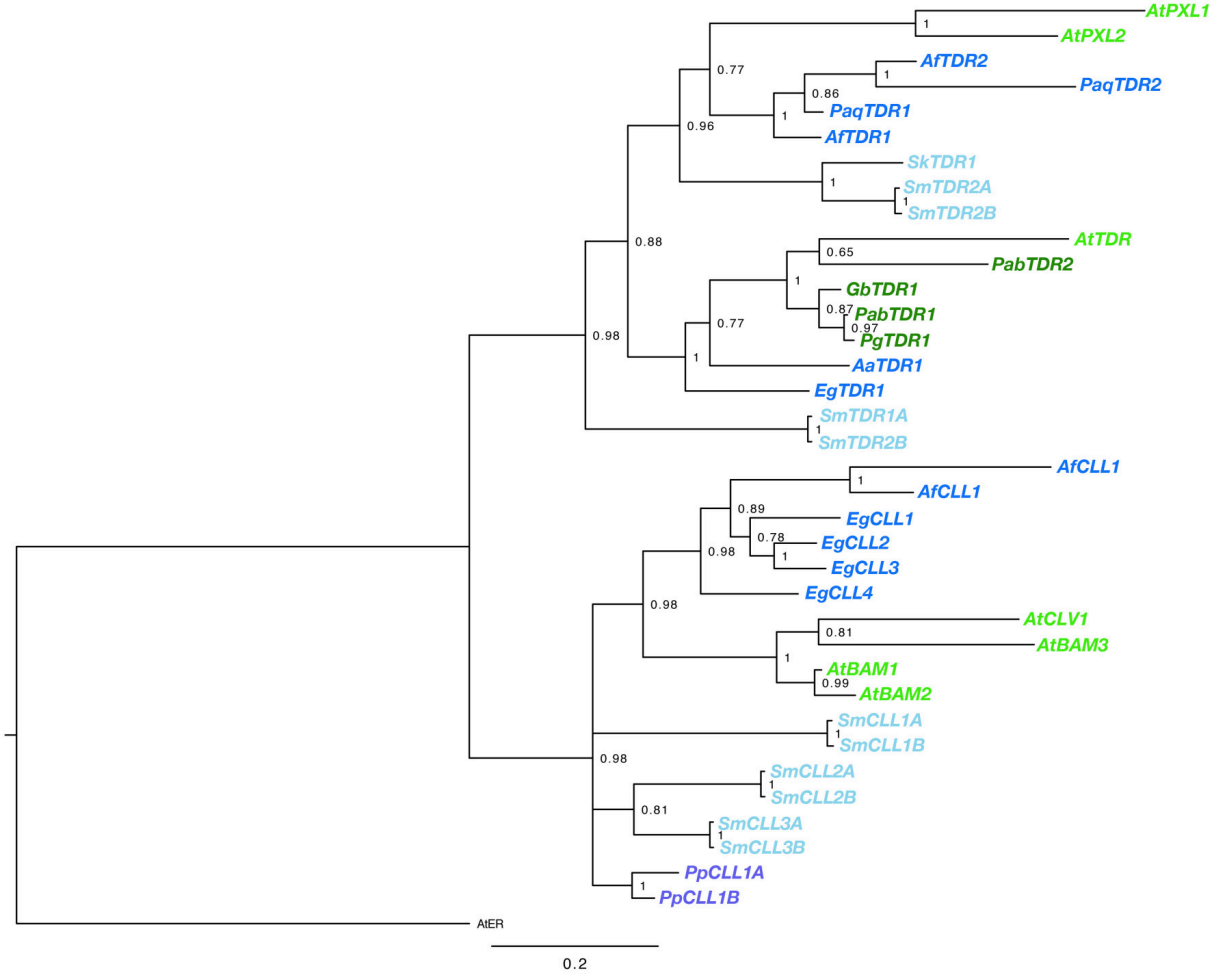
**Phylogenetic relationships of vascular plant TDR genes**. The phylogram was generated with the kinase domain sequences based on a Bayesian method. The posterior probabilities of trees are shown at the nodes. Using AtER as an outgroup, CLV and TDR genes each form monophyletic clades. Genes are color coded by taxon: moss, purple; lycophyte, light blue; ferns, dark blue; gymnosperm, dark green; angiosperm, light green. Species abbreviations are as follows: Pp, *Physcomitrella patens;* Sk, *Selaginella kraussiana;* Sm, *Selaginella moellendorfii;* Aa, *Adiantum aethiopicum;* Af, *Azolla filiculoides;* Eg, *Equisetum giganteum;* Paq, *Pteridium aquilinum;* Gb, *Ginkgo biloba;* Pab, *Picea abies;* Pg, *Picea glauca;* At, *Arabidopsis thaliana*. The paired *S. moellendorfii* sequences (A and B) are likely alleles.

BLAST searches using the kinase domain of *A. thaliana* TDR/PXY as a query, we found TDR sequences for gymnosperm and fern species from transcript databases. Sequences were obtained from *G. biloba, Azolla filiculoides, E. giganteum, Pteridium aquilinum* (Table S1). In addition, the *Sellaginella moellendorffii* genome contained four sequences highly similar to *AtTDR*, which are designated as *SmTDR1-A,B* and *SmTDR2-A,B* (Table S1; the pairs are two alleles). However, in *P. patens*, we could find no sequence highly similar to *AtTDR*, although orthologs of *AtCLV1*, a CLV3 receptor of *A. thaliana*, are encoded (*PpCLL1* and *PpCLL2* in Table S1; Miwa et al., 2009). CLL genes were also found in *A. filiculoides, E. giganteum*, and *S. moellendorffii* (Table S1). In addition, We obtained partial TDR sequences by application of degenerate PCR and RACE PCR to cDNA isolated from *G. biloba, A. aethiopicum* and *Sellaginella kraussiana* (Table S1). Kinase domains of the obtained sequences were aligned with the kinase domain sequences of the *ERECTA, CLV1*/*BAM, TDR*/*PXY*/*PXL* genes of *A. thaliana*. The phylogeny of the genes was reconstructed using a Bayesian method (Figure 2). Rooting the tree with the ERECTA/CLV1/BAM clade as an outgroup, vascular plant TDR genes form a highly supported monophyletic clade sister to a clade of land plant CLV1/BAM. The gene duplication producing the TDR/PXY and PXL clades predated the divergence of ferns from seed plants, with well-supported euphyllophyte clades for each of these gene classes. It seems likely that the gene duplication producing TDR and PXL genes occurred prior to the divergence of the lycophytes from the remainder of vascular plants, with *SmTDR2* being an ortholog of PXL and *SmTDR1* an ortholog of TDR, but the ambiguous position of *SmTDR1* precludes a definitive statement. Within the euphyllophyte TDR/PXY clade, phylogenetic relationships of the sequences largely mirror that of accepted euphyllophyte phylogeny. All gymnosperm sequences we identified are TDR orthologs, but broader sampling is required to determine whether the PXL ortholog was lost in gymnosperms. That *P. patens* genes are embedded, with high support, in the CLV1/BAM clade suggest that a TDR homolog was likely lost in the moss lineage.

### Effects of TDIF on vascular development in vascular plants

**Figure 3:**
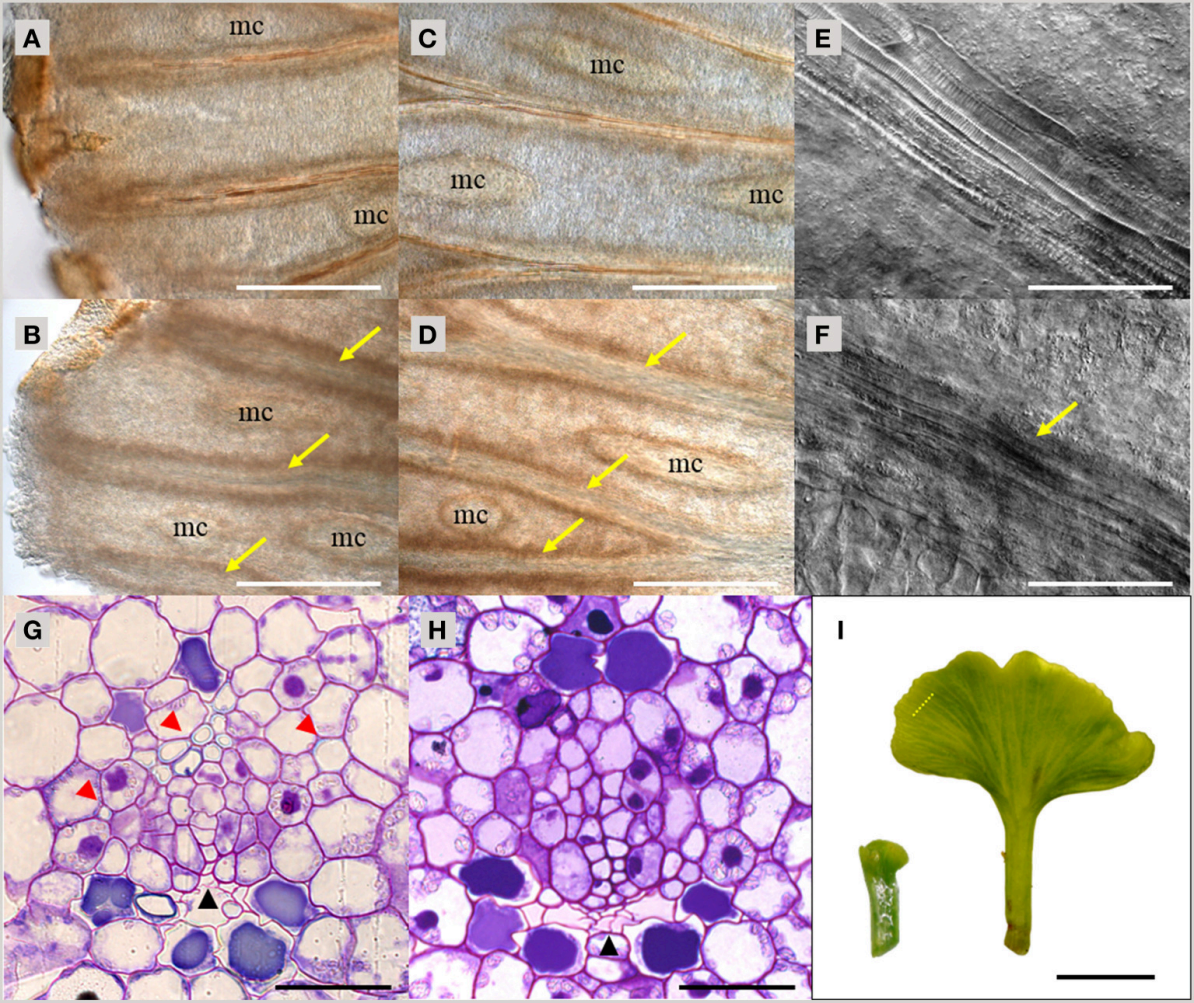
**Effects of TDIF treatment in ***Ginkgo biloba*** leaf**. **(A–H)** Veins of *G. biloba* leaves cultured for 10 days in liquid medium containing no additional peptide **(A,C,E,G)** or 10 μM TDIF **(B,D,F,H)**. **(A–F)** Images near the edge **(A,B)** or middle **(C–F)** of the leaf blade. Yellow arrows indicate veins without visible tracheids. Mucilage canals are indicated as mc. **(G,H)** Cross section of the veins. Black and red arrowheads indicate phloem and xylem, respectively. **(I)** Overall leaf morphology before (left) and after (right) 10-day culture. The dashed line illustrates the approximate position of sectioning plane for **(G,H)**. Scale bars: 500 μm in **(A–D)**, 100 μm in **(E,F)**, 50 μm in **(G,H),** and 0.5 cm in **(I)**.

As demonstrated above, TDIF and TDR orthologs are found throughout vascular plants. To investigate the function of TDIF/H-type CLE in vascular plants, we examined the bioactivity of peptide treatment in species from different taxa—gymnosperms, ferns and lycophytes. In *A. thaliana*, bioactivity of TDIF can be readily observed by liquid culturing of whole plants, thus we applied a similar approach in other species. Immature leaves on short shoots from a *G. biloba* tree were excised and grown in liquid culture for 10 days. In the vasculature, xylem differentiation occurs near the distal edge of the leaf blade and continuous xylem strands are formed along the veins (Figure 3A). TDIF treatment inhibited xylem differentiation, leading to veins developing without visible xylem tracheids even in the central region of the leaf blade (Figures 3A–D). In the veins without tracheids, elongated procambium-like cells are observed (Figures 3E,F). In cross-section, the loss of tracheids, as determined by secondary wall development (assessed by toluidine blue staining), in TDIF treated leaves was observed while phloem differentiation occurred normally, similar to the effects of exogenous bioactivity of TDIF in *A. thaliana* (Figures 3G,H; Hirakawa et al., 2008). The radius of the leaf blade grew from approximately 1.5–7.5 mm during this period (Figure 3I), and the overall growth was not affected by addition of 10 μM TDIF in the liquid medium.

**Figure 4:**
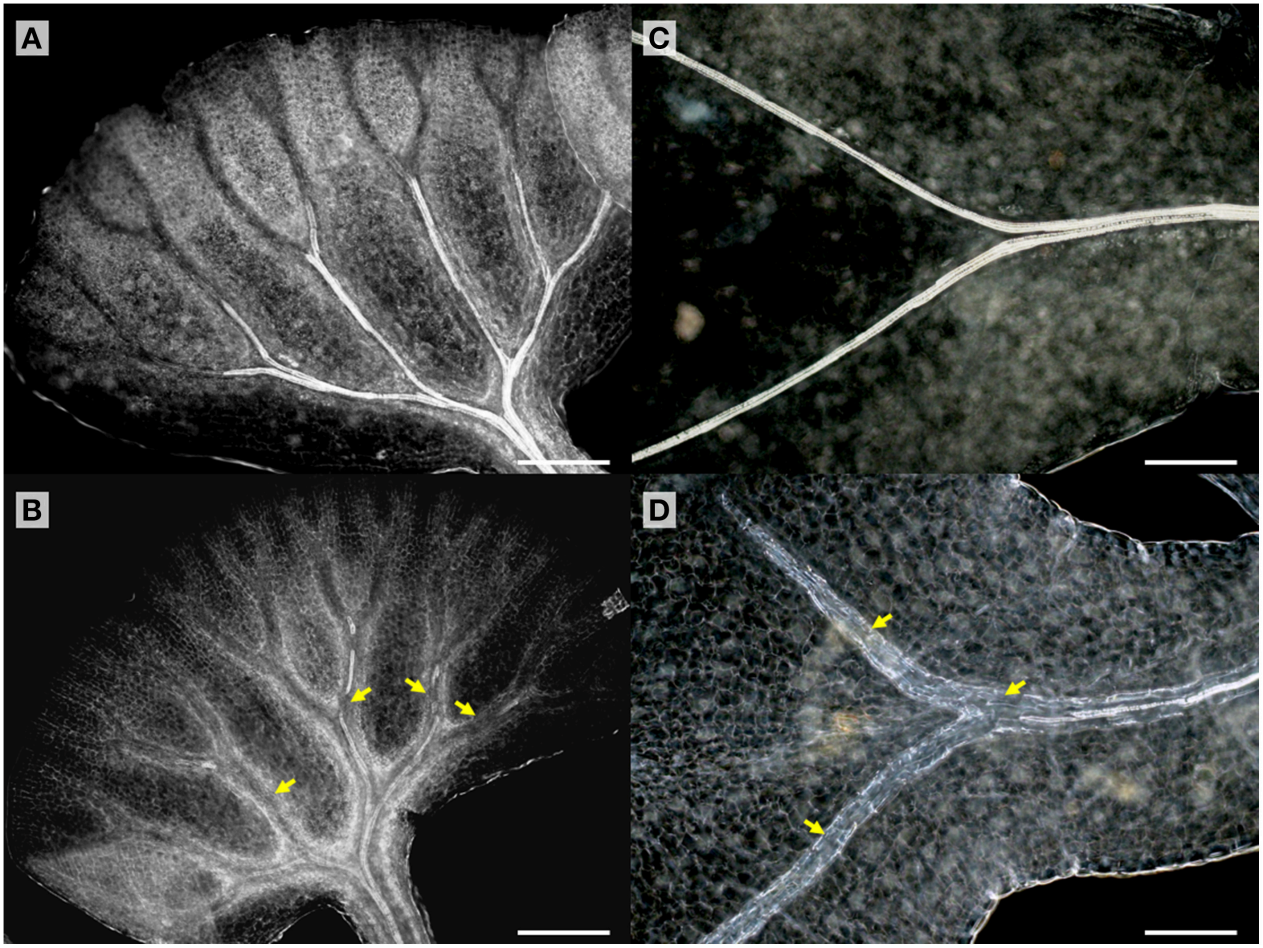
**Effects of TDIF treatment in fern fronds**. *Adiantum aethiopicum* **(A,B)** and *Asplenium* × *lucrosum* **(C,D)** fronds from plants cultured for 10 or 51 days in liquid medium containing no additional peptide **(A,C)** or 10 μM TDIF **(B,D)**. Yellow arrows indicate discontinued xylem strand in **(B)** and veins without visible tracheids in **(D)**. Scale bars: 200 μm.

TDIF sensitivity of *A. aethiopicum* was examined in similar experiments. Immature unfurled fronds were excised by cutting at the petiole and were grown in liquid medium. TDIF treatment reduced the formation of xylem strands in veins of fronds cultured for 10 days (Figures 4A,B). Although the inhibitory effect on xylem formation was not as strong as what was observed in *G. biloba* or *A. thaliana*, the discontinuous xylem formation indicates proper xylem cell differentiation was impeded by TDIF (Figure 4B). We further examined the effects of TDIF in *Asplenium* × *lucrosum* (*A. bulbiferum* × *A. dimorphum*) because this species produces many bulbils on its fronds, and bulbils can be cultured for a long period. All visible fronds were detached from bulbils and they were grown in liquid culture. A few fronds emerged in 17 days, after which the bulbils were transferred to TDIF containing media or control media and were further cultured for 34 days. In 51 day old plants treated with 10 μM TDIF, leaf veins without visible tracheids were observed (Figures 4C,D). Altogether, TDIF inhibits xylem cell differentiation in the two examined fern species.

**Figure 5:**
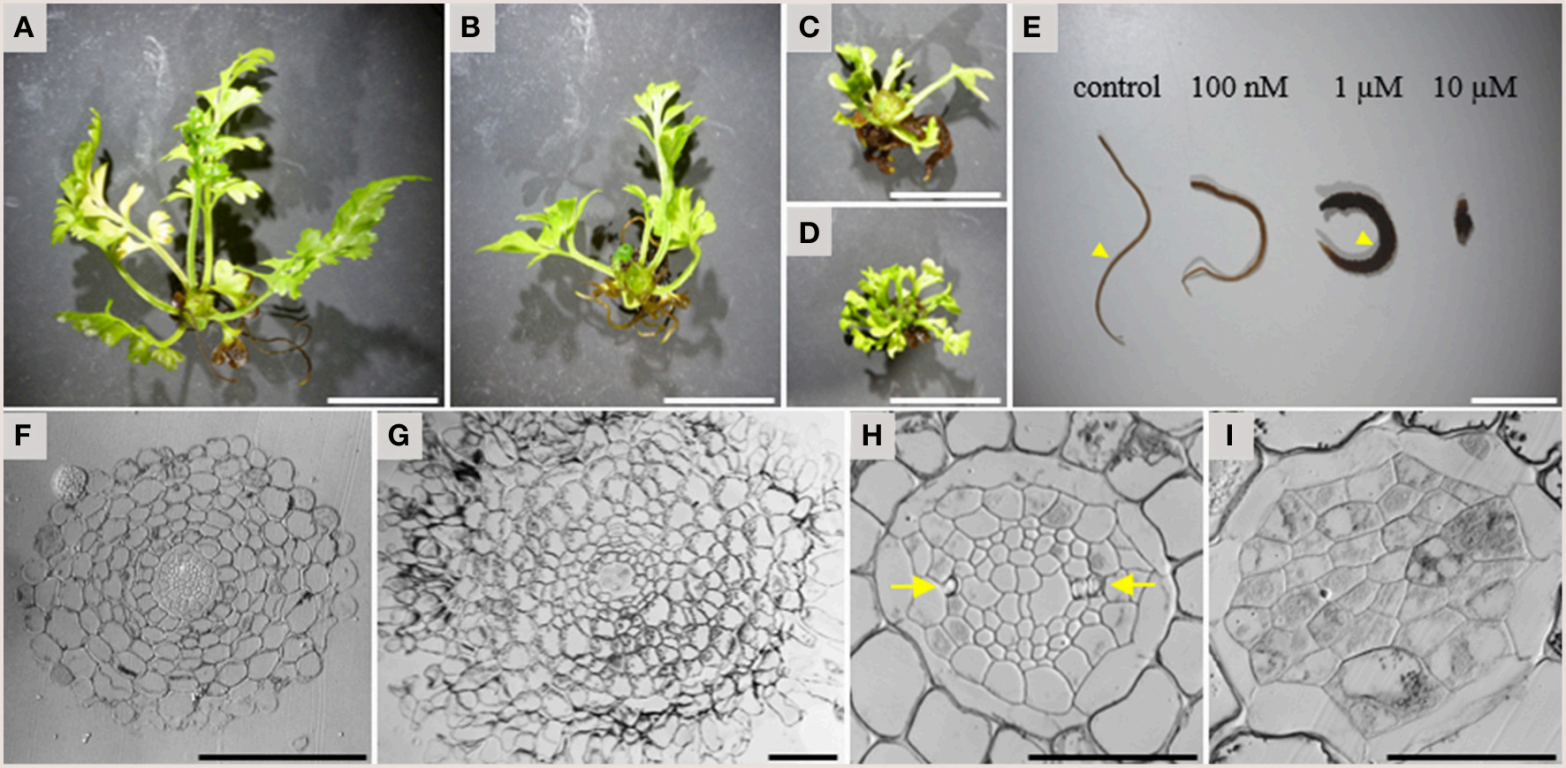
**Effects of TDIF on the morphology of ***Asplenium*** × ***lucrosum*****. **(A–D)** Overall morphology of *A*.× *lucrosum* plants grown for 3 months in liquid medium containing no additional peptide **(A)**, 100 nM TDIF **(B)**, 1 μM TDIF **(C),** or 10 μM TDIF **(D)**. **(E)** comparison of root morphology grown for 5 weeks in liquid culture containing different concentration of TDIF peptides as indicated. **(F–I)** Cross sections at the middle of the roots grown in control **(F,H)** or 1 μM TDIF **(G,I)** medium. Approximate positions for sectioning were illustrated in **(E)** by arrowheads. The images for **(H,I)** are magnification of central cylinder in **(F,G)**. Arrows in **(H)** indicate protoxylem poles. Scale bars: 2 cm in **(A–D)**, 1 cm in **(E)**, 100 μm in **(F,G)**, and 50 μm in **(H,I)**.

In addition to the inhibitory activity on xylem strand formation, TDIF had a strong inhibitory activity on overall plant growth in *A*. × *lucrosum*. After 3 months in culture, TDIF treatment reduced the growth in a dose dependent manner (Figures 5A–D). Although the size and complexity of fronds was reduced in TDIF treated plants, the number of fronds formed was increased. Root growth was also inhibited but the number of the roots formed increased. In addition, while root length was reduced, the thickness of roots increased (Figure 5E). In cross-section, roots grown with 1 μM TDIF had an increased number of cortex cell layers and abnormally shaped epidermal cells (Figures 5F,G). The size of the central vascular cylinder was not affected but its cellular organization was altered (Figures 5H,I). Under control conditions central vascular tissues are surrounded by one or two layers of pericycle cells. The vascular cylinder contains dipolar protoxylem tracheids, small phloem-like cells at the periphery, and relatively large cells near the center. In the peptide treatment central cylinders had a smaller number of relatively large cells without clear morphological features characteristic of differentiated vascular cells, indicating inhibition of proper cell differentiation and cell division.

**Figure 6:**
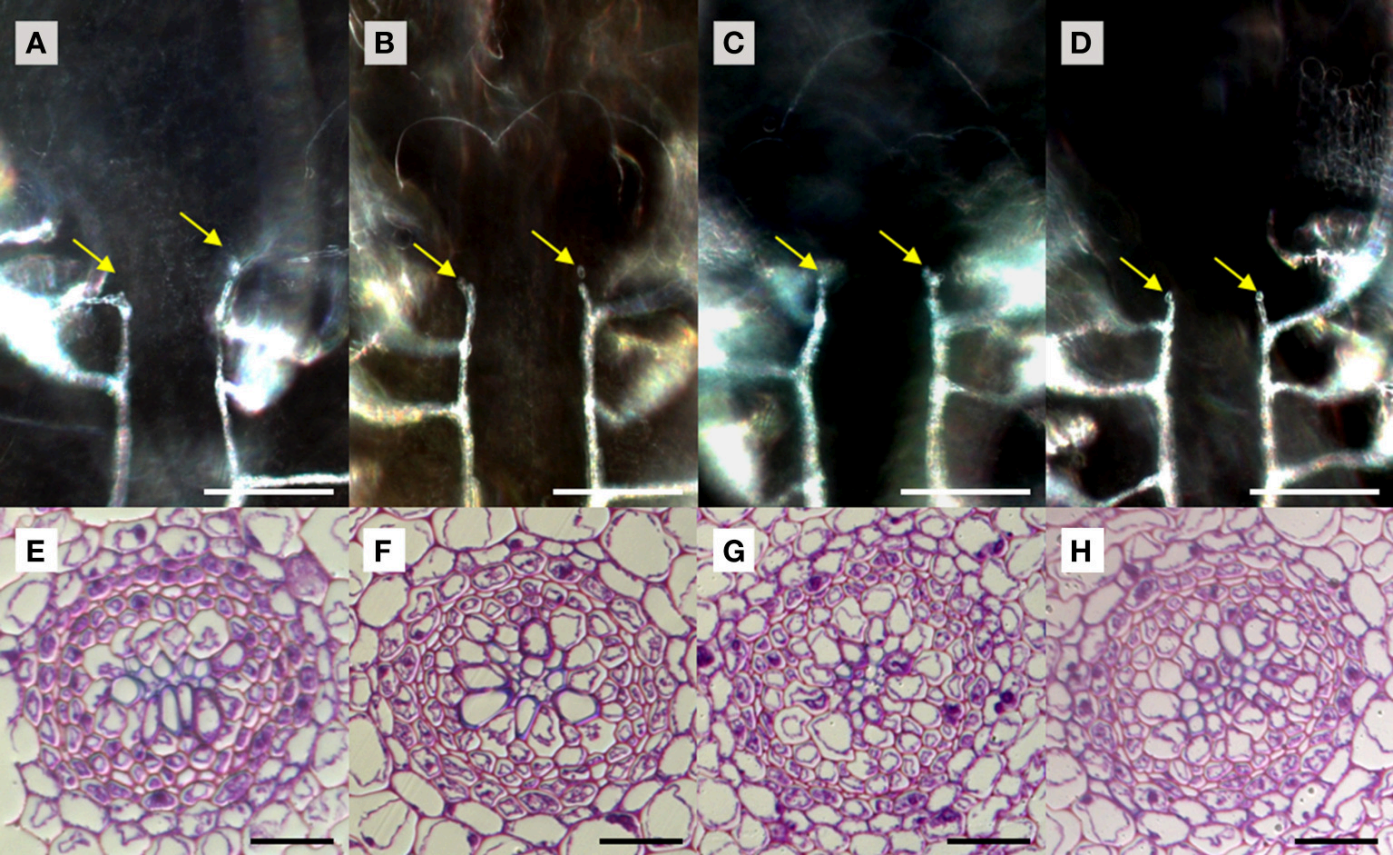
**Effects of TDIF in ***Selaginella kraussiana*****. Cleared whole-mount images of shoot meristem region with xylem strands **(A–D)** and cross sections of rhizophores **(E–H)** of *S. kraussiana* shoots grown for 3 weeks in liquid medium containing no additional peptide **(A,E)**, 5 μM TDIF **(B,F)**, 5 μM SkCLE1 peptide **(C,G)**, or 5 μM SKCLE1L peptide **(D,H)**. Yellow arrows in **(A–D)** indicate the termini of xylem strands (white lines in images) just below the shoot apical meristem. Scale bars: 100 μm **(A–D)**, 20 μm **(E–H)**.

We used *S. kraussiana* as a model to examine TDIF sensitivity in lycophytes. Excised shoots containing a pair of branched shoot tips were cultured 3 weeks in liquid culture with or without peptides. Near the shoot tips of control plants, two rows of xylem strands are formed, which are connected to xylem strands in leaves, following the pattern typical to the vascular development in lycophyte shoot (Figure 6A; Steeves and Sussex, 1989). In TDIF-treated shoots, the continuity of xylem strands as well as their relative position was not altered significantly—it was not affected by either SkCLE1 or SkCLE1L peptide (Figures 6A–D). Examining tissues other than the vasculature, we could not find any developmental defects by the peptides. We further examined the effect of peptides in rhizophores emerged during liquid culturing, but we did not see significant changes in rhizophore formation and growth due to peptide treatment. In cross-section of the *S. kraussiana* rhizophore the vascular cylinder contains central xylem and surrounding phloem tissues (Figure 6E). Tracheid differentiation was not suppressed in plants grown in the presence of 5 μM of any of the three peptides (Figures 6E–H). These data indicate that exogenously applied TDIF or SkCLE1 peptides do not have inhibitory activities on xylem cell differentiation in *S. kraussiana* although it is still not clear if the *SkCLE1* gene plays no role in xylem differentiation *in planta* because the lack of responses could be due to limitations of method as discussed later.

## Discussion

Molecular genetic studies have illustrated the importance of peptide signaling in communication between cells and tissues, which is essential for plant growth and development. An important question is how these signals are integrated into a specific developmental context, such as the formation of vasculature, during plant evolution. Comparative analyses on different plant taxa is one strategy to address this question. However, the availability of molecular genetic techniques is still limited to a small number of species. In this study, we analyzed the evolution of TDIF/CLE genes and the bioactivity of TDIF to examine their function in vascular development in taxa of the major clades of vascular plants including gymnosperms, ferns and lycophytes.

Phylogenetic analyses of sequences obtained in this study indicate that TDIF/H-type CLE genes and TDR receptor genes are conserved among vascular plants, suggesting that this signaling pathway may be active throughout the vascular plant lineage. In contrast, the moss *P. patens* lacks both TDIF/H-type CLE and TDR genes in its genome although it possesses R-type CLE genes. Further analyses on CLE family genes in other bryophytes, as well as charophycean algae, is necessary for understanding the origin and evolution of CLE peptide signaling.

TDIF treatment assays that TDIF is bioactive in shoot vascular tissues of gymnosperms and ferns. Inhibition of xylem strand differentiation in gymnosperm and fern species indicates conservation of the role for TDIF in tracheary element differentiation in euphyllophytes. In *A. thaliana*, TDIF signaling is implicated in the coordination of phloem and xylem differentiation from intervening procambium (Miyashima et al., 2013). As this type of vascular development, secondary xylem and phloem formation, is not active in extant ferns (Gifford and Foster, 1989; Spicer and Groover, 2010), the roles for TDIF in vascular development are not restricted to secondary vascular development. In the lycophyte *S. kraussiana*, we could not detect effects of TDIF on xylem differentiation in either shoots or rhizophores. Based on these observations, we propose that TDIF was integrated into shoot xylem differentiation in the euphyllophyte lineage after divergence from the lycophyte lineage. In this model, lycophytes and euphyllopytes may undergo a different process of xylem differentiation during vascular development. Future comparative analysis on the timing of xylem cell differentiation and the localization of TDIF signaling among vascular plant lineages is necessary to address if this model is valuable.

The peptide treatment assay in *A*. × *lucrosum* uncovered a novel developmental role for TDIF/H-type CLE. The strong growth inhibition observed by adding as low as 100 nM of TDIF was not observed in *A. thaliana*, rice or pine (Kinoshita et al., 2007; Strabala et al., 2014). In addition, TDIF did not merely reduce plant growth, but increased the production of lateral organs, increased root cortex cell layers and suppressed cell differentiation in the root vascular cylinder. Thus, TDIF signaling may confer multiple bioactivities in different contexts of tissue/organ development, which might reflect the different origins of lateral organs between seed plants and ferns.

There still exist non-trivial problems in the application of peptide treatment assays. Foremost, the gain-of-function effects caused by peptide treatment provide an idea of the potential for a peptide, which is not necessarily reflected in the role for intrinsic peptide signaling *in planta*. Development of specific agonist and antagonist is a future challenge to overcome this problem. Another problem is the efficacy of peptides. In this study, we did not see any alteration in vascular development of *S. kraussiana* by TDIF treatment, however the peptides might not be delivered to the vasculature, or alternatively, be degraded before reaching cell types expressing an appropriate receptor. In addition to the permeability problem, it is also possible that the native peptides possess side chain modifications, and thus the synthetic peptide does not represent the bioactivity of intrinsic peptides (Okamoto et al., 2013). Finally, a lack of a phenotype might reflect limited expression of the receptor. To overcome these problems, establishing experimental systems such as transformation techniques or cell culture systems is also important.

### Conflict of interest statement

The authors declare that the research was conducted in the absence of any commercial or financial relationships that could be construed as a potential conflict of interest.

## References

[B1] Araya T. Miyamoto M. Wibowo J. Suzuki A. Kojima S. Tsuchiya Y. N.. (2014). CLE-CLAVATA1 peptide-receptor signaling module regulates the expansion of plant root systems in a nitrogen-dependent manner. Proc. Natl. Acad. Sci. U.S.A. 111, 2029–2034. 10.1073/pnas.1319953111 24449877 PMC3918772

[B2] Brand U. Fletcher J. C. Hobe M. Meyerowitz E. M. Simon R. (2000). Dependence of stem cell fate in Arabidopsis on a feedback loop regulated by CLV3 activity. Science 289, 617–619. 10.1126/science.289.5479.617 10915624

[B3] Clark S. E. Running M. P. Meyerowitz E. M. (1995). CLAVATA3 is a specific regulator of shoot and floral meristem development affecting the same processes as CLAVATA1. Development 121, 2057–2067.

[B4] Depuydt S. Rodriguez-Villalon A. Santuari L. Wyser-Rmili C. Ragni L. Hardtke C. S. (2013). Suppression of Arabidopsis protophloem differentiation and root meristem growth by CLE45 requires the receptor-like kinase BAM3. Proc. Natl. Acad. Sci. U.S.A. 110, 7074–7079. 10.1073/pnas.1222314110 23569225 PMC3637694

[B5] de Vries J. Fischer A. M. Roettger M. Rommel S. Schluepmann H. Bräutigam A.. (2015). Cytokinin-induced promotion of root meristem size in the fern Azolla supports a shoot-like origin of euphyllophyte roots. New Phytol. [Epub ahead of print]. 10.1111/nph.13630 26358624 PMC5049668

[B6] DeYoung B. J. Bickle K. L. Schrage K. J. Muskett P. Patel K. Clark S. E. (2006). The CLAVATA1-related BAM1, BAM2 and BAM3 receptor kinase-like proteins are required for meristem function in Arabidopsis. Plant J. 45, 1–16. 10.1111/j.1365-313X.2005.02592.x 16367950

[B7] Endo S. Shinohara H. Matsubayashi Y. Fukuda H. (2013). A novel pollen-pistil interaction conferring high-temperature tolerance during reproduction via CLE45 signaling. Curr. Biol. 23, 1670–1676. 10.1016/j.cub.2013.06.060 23910659

[B8] Etchells J. P. Turner S. R. (2010). The PXY-CLE41 receptor ligand pair defines a multifunctional pathway that controls the rate and orientation of vascular cell division. Development 137, 767–774. 10.1242/dev.044941 20147378

[B9] Fisher K. Turner S. (2007). PXY, a receptor-like kinase essential for maintaining polarity during plant vascular-tissue development. Curr. Biol. 17, 1061–1066. 10.1016/j.cub.2007.05.049 17570668

[B10] Fiume E. Fletcher J. C. (2012). Regulation of Arabidopsis embryo and endosperm development by the polypeptide signaling molecule CLE8. Plant Cell 24, 1000–1012. 10.1105/tpc.111.094839 22427333 PMC3336133

[B11] Fletcher J. C. Brand U. Running M. P. Simon R. Meyerowitz E. M. (1999). Signaling of cell fate decisions by CLAVATA3 in Arabidopsis shoot meristems. Science 283, 1911–1914. 10.1126/science.283.5409.1911 10082464

[B12] Gelman A. Rubin D. B. (1992). Inference from iterative simulation using multiple sequences. Stat. Sci. 7, 457–511. 10.1214/ss/1177011136

[B13] Gifford E. M. Foster A. S. (1989). Morphology and Evolution of Vascular Plants, 3rd Edn. New York, NY: W.H. Freeman and Co.

[B14] Hirakawa Y. Kondo Y. Fukuda H. (2010). TDIF peptide signaling regulates vascular stem cell proliferation via the WOX4 homeobox gene in Arabidopsis. Plant Cell 22, 2618–2629. 10.1105/tpc.110.076083 20729381 PMC2947162

[B15] Hirakawa Y. Shinohara H. Kondo Y. Inoue A. Nakanomyo I. Ogawa M.. (2008). Non-cell-autonomous control of vascular stem cell fate by a CLE peptide/receptor system. Proc. Natl. Acad. Sci. U.S.A. 105, 15208–15213. 10.1073/pnas.0808444105 18812507 PMC2567516

[B16] Huelsenbeck J. P. Ronquist F. (2001). MRBAYES: Bayesian inference of phylogenetic trees. Bioinformatics 17, 754–755. 10.1093/bioinformatics/17.8.754 11524383

[B17] Ito Y. Nakanomyo I. Motose H. Iwamoto K. Sawa S. Dohmae N.. (2006). Dodeca-CLE peptides as suppressors of plant stem cell differentiation. Science 313, 842–845. 10.1126/science.1128436 16902140

[B18] Jun J. H. Fiume E. Fletcher J. C. (2008). The CLE family of plant polypeptide signaling molecules. Cell. Mol. Life Sci. 65, 743–755. 10.1007/s00018-007-7411-5 18034320 PMC11131854

[B19] Kinoshita A. Nakamura Y. Sasaki E. Kyozuka J. Fukuda H. Sawa S. (2007). Gain-of-function phenotypes of chemically synthetic CLAVATA3/ESR-related (CLE) peptides in *Arabidopsis thaliana* and *Oryza sativa*. Plant Cell Physiol. 48, 1821–1825. 10.1093/pcp/pcm154 17991631

[B20] Kondo T. Sawa S. Kinoshita A. Mizuno S. Kakimoto T. Fukuda H.. (2006). A plant peptide encoded by CLV3 identified by *in situ* MALDI-TOF MS analysis. Science 313, 845–848. 10.1126/science.1128439 16902141

[B21] Kondo Y. Hirakawa Y. Kieber J. J. Fukuda H. (2011). CLE peptides can negatively regulate protoxylem vessel formation via cytokinin signaling. Plant Cell Physiol. 52, 37–48. 10.1093/pcp/pcq129 20802224 PMC3023848

[B22] Matsubayashi Y. (2014). Posttranslationally modified small-peptide signals in plants. Annu. Rev. Plant Biol. 65, 385–413. 10.1146/annurev-arplant-050312-120122 24779997

[B23] Miwa H. Tamaki T. Fukuda H. Sawa S. (2009). Evolution of CLE signaling: origins of the CLV1 and SOL2/CRN receptor diversity. Plant Signal. Behav. 4, 477–481. 10.4161/psb.4.6.8391 19816140 PMC2688290

[B24] Miyashima S. Sebastian J. Lee J. Y. Helariutta Y. (2013). Stem cell function during plant vascular development. EMBO J. 32, 178–193. 10.1038/emboj.2012.301 23169537 PMC3553377

[B25] Oelkers K. Goffard N. Weiller G. F. Gresshoff P. M. Mathesius U. Frickey T. (2008). Bioinformatic analysis of the CLE signaling peptide family. BMC Plant Biol. 8:1. 10.1186/1471-2229-8-1 18171480 PMC2254619

[B26] Ogawa M. Shinohara H. Sakagami Y. Matsubayashi Y. (2008). Arabidopsis CLV3 peptide directly binds CLV1 ectodomain. Science 319, 294. 10.1126/science.1150083 18202283

[B27] Ogawa-Ohnishi M. Matsushita W. Matsubayashi, Y. (2013). Identification of three hydroxyproline O-arabinosyltransferases in *Arabidopsis thaliana*. Nat. Chem. Biol. 9, 726–730. 10.1038/nchembio.1351 24036508

[B28] Ohyama K. Shinohara H. Ogawa-Ohnishi M. Matsubayashi Y. (2009). A glycopeptide regulating stem cell fate in *Arabidopsis thaliana*. Nat. Chem. Biol. 5, 578–580. 10.1038/nchembio.182 19525968

[B29] Okamoto S. Ohnishi E. Sato S. Takahashi H. Nakazono M. Tabata S.. (2009). Nod factor/nitrate-induced CLE genes that drive HAR1-mediated systemic regulation of nodulation. Plant Cell Physiol. 50, 67–77. 10.1093/pcp/pcn194 19074184

[B30] Okamoto S. Shinohara H. Mori T. Matsubayashi Y. Kawaguchi M. (2013). Root-derived CLE glycopeptides control nodulation by direct binding to HAR1 receptor kinase. Nat. Commun. 4, 2191. 10.1038/ncomms3191 23934307

[B31] Schoof H. Lenhard M. Haecker A. Mayer K. F. Jürgens G. Laux T. (2000). The stem cell population of Arabidopsis shoot meristems in maintained by a regulatory loop between the CLAVATA and WUSCHEL genes. Cell 100, 635–644. 10.1016/S0092-8674(00)80700-X 10761929

[B32] Shinohara H. Moriyama Y. Ohyama K. Matsubayashi Y. (2012). Biochemical mapping of a ligand-binding domain within Arabidopsis BAM1 reveals diversified ligand recognition mechanisms of plant LRR-RKs. Plant J. 70, 845–854. 10.1111/j.1365-313X.2012.04934.x 22321211

[B33] Spicer R. Groover A. (2010). Evolution of development of vascular cambia and secondary growth. New Phytol. 186, 577–592. 10.1111/j.1469-8137.2010.03236.x 20522166

[B34] Steeves T. A. Sussex I. M. (1989). Patterns in Plant Development, 2nd Edn. Cambridge: Cambridge University Press.

[B35] Strabala T. J. O'donnell P. J. Smit A. M. Ampomah-Dwamena C. Martin E. J. Netzler N.. (2006). Gain-of-function phenotypes of many CLAVATA3/ESR genes, including four new family members, correlate with tandem variations in the conserved CLAVATA3/ESR domain. Plant Physiol. 140, 1331–1344. 10.1104/pp.105.075515 16489133 PMC1435808

[B36] Strabala T. J. Phillips L. West M. Stanbra L. (2014). Bioinformatic and phylogenetic analysis of the CLAVATA3/EMBRYO-SURROUNDING REGION (CLE) and the CLE-LIKE signal peptide genes in the Pinophyta. BMC Plant Biol. 14:47. 10.1186/1471-2229-14-47 24529101 PMC4016512

[B37] Tamaki T. Betsuyaku S. Fujiwara M. Fukao Y. Fukuda H. Sawa S. (2013). SUPPRESSOR OF LLP1 1-mediated C-terminal processing is critical for CLE19 peptide activity. Plant J. 76, 970–981. 10.1111/tpj.12349 24118638

[B38] Vanneste K. Sterck L. Myburg A. A. Van de Peer Y. Mizrachi E. (2015). Horsetails are ancient polyploids: evidence from *Equisetum giganteum*. Plant Cell 27, 1567–1578. 10.1105/tpc.15.00157 26002871 PMC4498207

[B39] Whitford R. Fernandez A. De Groodt R. Ortega E. Hilson P. (2008). Plant CLE peptides from two distinct functional classes synergistically induce division of vascular cells. Proc. Natl. Acad. Sci. U.S.A. 105, 18625–18630. 10.1073/pnas.0809395105 19011104 PMC2587568

